# Lipid-Lowering Bioactivity of Microalga *Nitzschia laevis* Extract Containing Fucoxanthin in Murine Model and Carcinomic Hepatocytes

**DOI:** 10.3390/ph14101004

**Published:** 2021-09-29

**Authors:** Bingbing Guo, Yonghui Zhou, Bin Liu, Yongjin He, Feng Chen, Ka-Wing Cheng

**Affiliations:** 1 Faculty of Environment and Life, Beijing University of Technology, 100 Pingleyuan, Chaoyang District, Beijing 100124, China; guobingbing@bjut.edu.cn (B.G.); zhouyh011@emails.bjut.edu.cn (Y.Z.); 2 Shenzhen Key Laboratory of Marine Microbiome Engineering, Institute for Advanced Study, Shenzhen University, Nanshan District, Shenzhen 518060, China; liubin@szu.edu.cn; 3 Institute for Innovative Development of Food Industry, Shenzhen University, Shenzhen 518060, China; 4 College of Life Sciences, Fujian Normal University, No.1, Keji Road, Fuzhou 350117, China; yongjinhe@fjnu.edu.cn

**Keywords:** microalgal extract, fucoxanthin, lipid-lowering bioactivity, non-alcoholic fatty liver disease

## Abstract

Non-alcoholic fatty liver disease (NAFLD), characterized by hepatic steatosis, is one of the most common liver diseases worldwide. So far, no definitive medical treatment has been established to treat NAFLD except for lifestyle medication. *Nitzschia laevis* extract (NLE), a microalgal extract rich in fucoxanthin, has been previously demonstrated to reduce bodyweight in high-fat-diet (HFD) C57BL/6J mice, indicating potential for prevention of NAFLD. In the present study, we investigated the lipid-lowering effects of NLE in HFD-induced steatosis murine model and palmitate-treated HepG2 cells. The results showed that NLE significantly lowered inguinal fat and attenuated hepatic steatosis in C57BL/6J mice. Especially, NLE significantly prevented lipid accumulation in HepG2 cells. This was probably due to its capability to enhance hepatic mitochondrial function as evidenced by the increased oxygen consumption rate (OCR) and mitochondrial membrane potential (MMP), and repress fatty acid synthesis through phosphorylation of acetyl-CoA carboxylase (ACC). Moreover, fucoxanthin was identified to be responsible for the lipid-lowering effect of NLE. Taken together, NLE or other microalgal fucoxanthin-rich products are promising natural products that may help prevent against NAFLD.

## 1. Introduction

Non-alcoholic fatty liver disease (NAFLD) has become a major health problem globally with an estimated prevalence of 30% and it is expected to continue increasing in parallel with metabolic abnormalities including obesity and diabetes [1,2]. Characterized by excessive hepatic lipid accumulation, NAFLD may progress from simple steatosis to non-alcoholic steatohepatitis (NASH), liver fibrosis and finally hepatocellular carcinoma, if appropriate interventions are not applied. Therefore, prevention and/or reverse of overaccumulation of lipids in hepatocytes remains a plausible strategy for the management of NAFLD.

In recent years, dietary phytochemicals or phytochemical-rich plant extracts have been increasingly appreciated as effective agents for alleviation of fatty liver disease [3,4]. Among the phytochemical candidates, carotenoids, owing to their generally hydrophobic nature, have exhibited promising efficacy in preclinical models of NAFLD and NASH [5]. For instance, lycopene was reported to attenuate lipid accumulation in human liver cell lines and prevent the development of high-fat-diet (HFD) induced NASH in rodents [3]. Astaxanthin, a marine carotenoid well-known for its potent antioxidant capacity, effectively prevented hepatic lipid accumulation by down-regulating lipogenic genes and improved mitochondrial oxidative metabolism by protecting mitochondrial redox homeostasis and functional integrity against oxidative stress [6]. *Nitzschia laevis* extract (NLE) was found in our previous study to reduce lipid content of C57BL/6J mice [7]. However, the underlying mechanisms for the hepatic lipid-lowering effect of NLE has not yet to be characterized. Meanwhile, fucoxanthin, a major carotenoid in NLE, is known for its anti-obesity effect likely via stimulating lipid metabolism in adipose tissue [7]. It was also found that fucoxanthin inhibited hepatic fat accumulation in mice fed with a high-fat diet and murine hepatocyte Hepa1–6 cells [8]. Thus, it is reasonable to hypothesize that NLE might exhibit good lipid-lowering activity.

Usually, NAFLD easily causes mitochondrial dysfunction, which may include decreased mitochondrial membrane potential (MMP) and down-regulated oxidative phosphorylation [9]. This in turn would further contribute to progression of the disease due to impaired mitochondrial fatty acid metabolism [10]. It has been reported that improving mitochondrial function could help to prevent hepatic lipid accumulation [3]. Acetyl-CoA carboxylase (ACC), the rate-limiting enzyme of fatty acid biosynthesis, plays a critical role in hepatic lipid metabolism. In particular, insulin-mediated dephosphorylation increases its activity, while glucagon-mediated phosphorylation decreases it. Studies have found that inhibition of ACC through phosphorylation could inhibit lipogenesis in rat hepatocytes, which is supposed to be a promising approach to the prevention of hepatic lipid accumulation [11,12]. 

The current study evaluated the lipid-lowering effects of NLE in both HFD-induced steatosis C57BL/6J mice and palmitate-treated HepG2 cell lines. The underlying mechanisms were further investigated using HepG2 cells. Mitochondrial lipid metabolism was partially characterized by analyzing mitochondrial oxygen consumption rate (OCR) and MMP. Expression of ACC was also determined to point fatty acids synthesis changes in hepatocytes upon treatment with NLE. Furthermore, pure fucoxanthin was utilized to corroborate its role in the lipid-lowering activity of NLE. 

## 2. Results

### 2.1. NLE Composition and Fucoxanthin Identification

**Figure 1:**
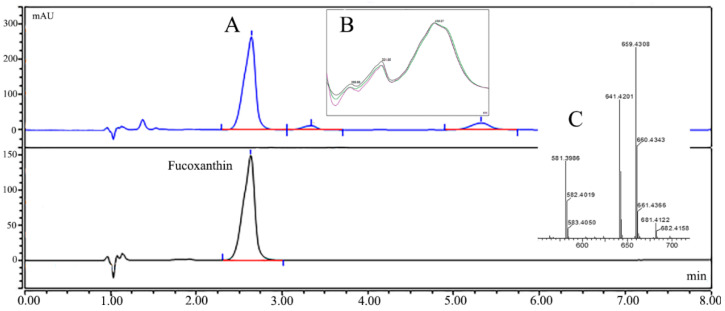
Identification of fucoxanthin in NLE. (**A**) HPLC result of NLE and the peak of fucoxanthin; (**B**,**C**) UV absorption and mass data of the peak of fucoxanthin.

Chemical analysis revealed that NLE contains a mixture of bio-compounds like carotenoids, polyphenols, and polyunsaturated fatty acids [7]. Fucoxanthin was found to be the major (75.3%) carotenoids in NLE and it was identified and confirmed by chromatography and mass spectrometry methods (Figure 1).

### 2.2. NLE Prevented Bodyweight Gain and Lipid Accumulation in HFD-Treated Mice

**Figure 2:**
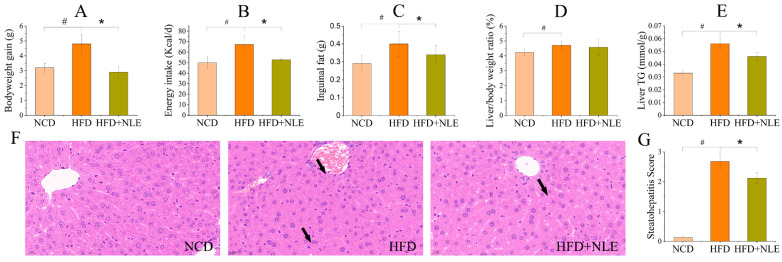
Effects of NLE on bodyweight gain and lipid content of HFD-treated mice. (**A**) Bodyweight gain of mice in different groups; (**B**) Energy intake of mice in different groups; (**C**) Inguinal fat content of mice in different groups; (**D**) Liver weight in of mice in different groups; (**E**) Liver TG content in all groups; (**F**,**G**) H&E staining results of the liver and the steatohepatitis score in all groups. The dark arrows indicate steatosis. NCD: Normal chow diet; HFD: High fat diet; HFD + NLE: High fat diet + *Nitzschia laevis* extract (100 mg/kg/d). # indicates significantly different at *p* < 0.05 compared with NCD. * indicates significantly different at *p* < 0.05 compared with HFD.

HFD treatment for 12 weeks significantly increased bodyweight gain and induced steatosis in C57BL/6J mice (Figure 2). With NLE supplementation, the bodyweight gain of mice fed on HFD was comparable to that of mice in NCD group (Figure 2A). The energy intake was decreased accordingly in HFD + NLE group (Figure 2B). The inguinal fat, a typical indicator for body fat, was greatly reduced by NLE, indicating lipid content reduction in HFD-induced mice (Figure 2C). In addition, liver weight was significantly increased through HFD feeding, and NLE showed a trend to reduce it (Figure 2D), as well as the total triacylglycerol (TG) content (Figure 2E). Impressively, hematoxylin-eosin (H&E) staining of the liver showed severe microbubble steatosis in HFD mice, and this was significantly attenuated by NLE supplementation (Figure 2F,G). No inflammatory cell infiltration was found in the liver during the experimental period. Meanwhile, the biochemical analysis also showed NLE reduced serum lipid contents that were induced by HFD (Table 1).

### 2.3. NLE Attenuated Lipid Accumulation in HepG2 Cells

**Figure 3:**
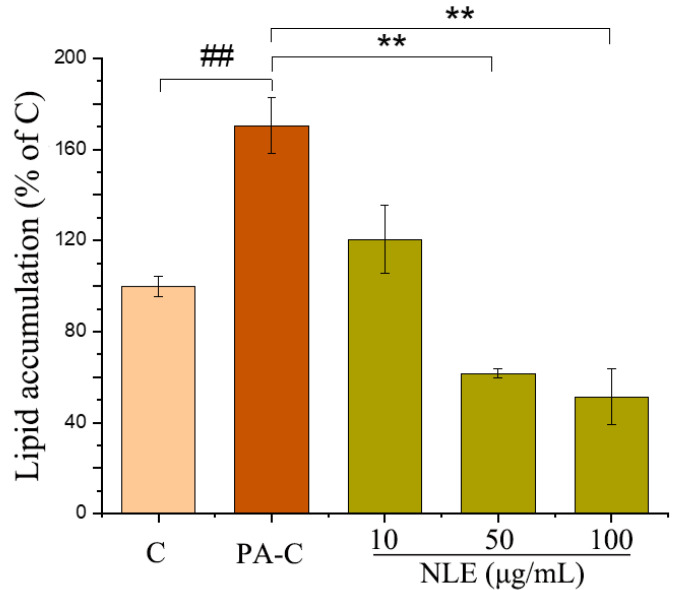
Effect of different concentrations of NLE on lipid accumulation in HepG2 cells. The data represent means ± SD of three independent experiments. C: Control; PA-C: Control+ palmitate; NLE: *Nitzschia laevis* extract. ## indicates significantly different at *p* < 0.01 compared with C. ** indicates significantly different at *p* < 0.01 compared with PA-C.

For further investigation of the lipid-lowering bioactivity of NLE, palmitate-induced HepG2 cells were applied. MTT assay indicates that the tested concentrations of NLE (10, 50 and 100 µg/mL) did not exhibit any appreciable cytotoxicity in the HepG2 cell lines under the experimental conditions (data not shown). As expected, palmitate (PA) increased lipid content of the cells by nearly 70% relative to vehicle control (Figure 3). Notably, even the lowest concentration (10 µg/mL) of the three tested NLE treatments was able to almost completely abrogate the effect of palmitate on cellular lipid accumulation. This was further manifested by the protective effect of the two higher concentrations which potently reduced the lipid content of the cells to a level that was ~50% lower than the vehicle control. 

### 2.4. NLE Increased Mitochondrial Fatty Acids Metabolism in HepG2 Cells

**Figure 4:**
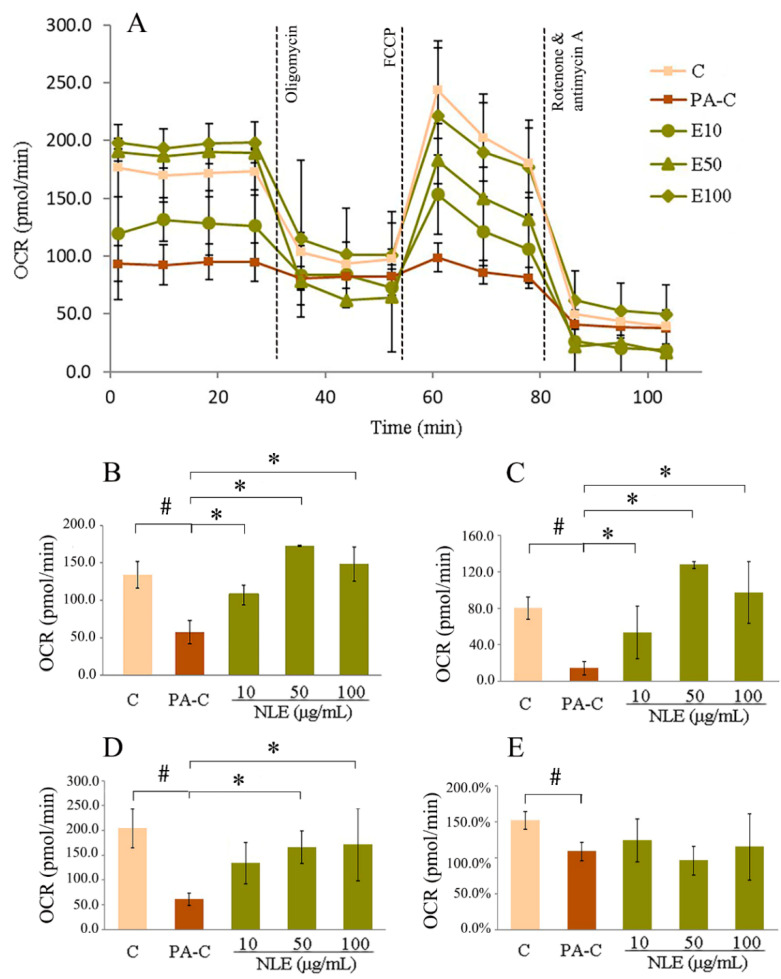
NLE increased oxygen consumption rate (OCR) of HepG2 cells. (**A**) Kinetics of OCR in HepG2 cells treated with different concentrations of NLE; (**B**) baseline respiration; (**C**) ATP-linked respiration; (**D**) maximal respiration and (**E**) spare respiratory capacity (%). Data are presented as means ± SD from three independent experiments. C: Control; PA-C: Control+ palmitate; NLE: *Nitzschia laevis* extract. E10, E50 and E100: NLE at the concentration of 10, 50 and 100 µg/mL. # indicates significantly different at *p* < 0.05 compared with C. * indicates significantly different at *p* < 0.05 compared with PA-C.

To test whether mitochondrial respiration contributed to the reduction of lipid accumulation in hepatocytes caused by NLE, OCR was measured for the different experimental groups. Incubation with PA for 12 h greatly reduced mitochondrial metabolism of HepG2 cells as supported by their lower basal respiration, ATP-linked respiration, maximal respiration and, spare respiratory capacity compared with control (Figure 4). Basal oxygen consumption was generally improved by NLE (Figure 4A,B). A maximum OCR of 172.6 pmol/min was observed with 50 µg/mL of NLE. After adding oligomycin, an inhibitor of H^+^-ATP-synthase, ATP-linked respiration/OCR decreased considerably (Figure 4A,C). In particular, 50 and 100 µg/mL NLE not only completely abrogated the suppressive effect of PA on ATP-linked respiration in HepG2 cells but it actually showed a net stimulatory effect, as evidenced by the higher levels of OCR than the vehicle control (especially at 50 µg/mL). This strongly indicates that NLE could enhance mitochondrial metabolism of fatty acids. Afterwards, the uncoupling agent carbonyl cyanide-4 (trifluoromethoxy) phenylhydrazone (FCCP) was used to dissipate the MMP, thus driving the electron transport chain to function at its maximal rate. As shown in Figure 4A,D, NLE recovered the PA-induced suppression of maximal respiration in a dose-dependent manner. Finally, the electron transport chain was completely inhibited by Rotenone and antimycin A and no significant changes in the spare respiratory capacity were observed with NLE treatments.

### 2.5. NLE Increased Mitochondrial Membrane Potential of HepG2 Cells

**Figure 5:**
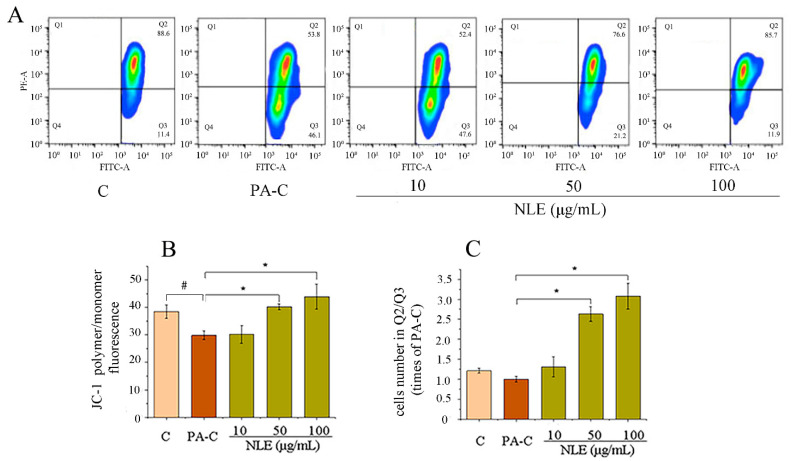
NLE increased mitochondrial membrane potential in HepG2 cells. (**A**) FACS analysis of cells stained with JC-1 for all groups; (**B**) the ratio of polymer (red)/monomer (green) fluorescence; (**C**) ratio of the cell numbers between Q2 and Q3. Data are presented as means ± SD from three independent experiments. C: control; PA-C: Control+ palmitate, NLE: *Nitzschia laevis* extract. # indicates significantly different at *p* < 0.05 compared with C. * indicates statistically significant at *p* < 0.05 compared with PA-C.

To obtain further evidence for the stimulation of lipid metabolism, mitochondrial function which was indicated by MMP was measured by JC-1 staining and flow cytometry (Figure 5). At high MMP, JC-1 forms J-aggregates in mitochondria emitting red fluorescence; while JC-1 exists as a monomeric green-emitting form when MMP is low. The polymer/monomer ratio, which was reduced by PA treatment, was recovered by NLE (Figure 5A,B), indicating an improvement of MMP. PA treatment led to significant increase in the percentage of the cells clustered in quadrant 3 (Q3) and a concomitant decrease in the percentage of cells in Q2, indicating a greatly reduced MMP compared to the control. Remarkably, the two higher doses of NLE were able to counteract the effect of PA by dose-dependently decreasing the number of cells in Q3 and increasing the number of cells in Q2 (Figure 5A,C). Of note, at 100 µg/mL, NLE completely normalized the ratio of cells in Q2/Q3. These data support that NLE is highly effective in protecting mitochondrial function, showing potential for the improvement of lipid metabolism.

### 2.6. NLE Inhibited Fatty Acid Synthesis in HepG2 Cells

**Figure 6:**
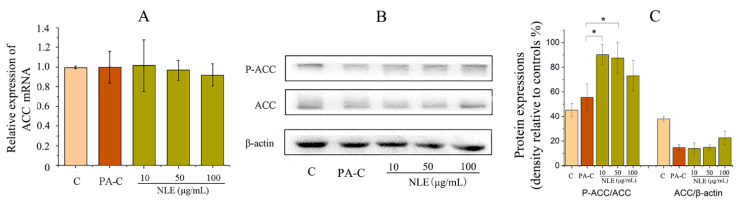
Analysis of genes related to lipid metabolism in HepG2 cells. (**A**) Relative expression of ACC mRNA; (**B**,**C**) western blot analysis of lysates of HepG2 cells treated with different concentrations of NLE and densitometry of immunoblots normalized to β-actin. Data are presented as means ± SD from three independent experiments. C: control; PA-C: Control + palmitate, NLE: *Nitzschia laevis* extract. * indicates significantly different at *p* < 0.05 compared with PA-C.

Having observed that NLE prevented PA-induced lipid accumulation and promoted lipid metabolism through improving mitochondrial function in HepG2 cells, expression of ACC, the fatty acid synthesis rate-limiting enzyme, was subsequently examined to better understand whether NLE also modulates fatty acid synthesis. Neither of PA-C and NLE treatments seemed to have any significant impact on the mRNA expression of ACC (Figure 6A). In addition, NLE did not have any significant impact on the expression level of ACC (Figure 6B). However, it significantly upregulated the phosphorylation of ACC (*p*-ACC), which likely contributed to the attenuated PA-induced fatty acid biosynthesis (Figure 6B,C). 

### 2.7. Fucoxanthin Prevented Hepatic Lipid Accumulation in PA-Treated HepG2 Cells

**Figure 7:**
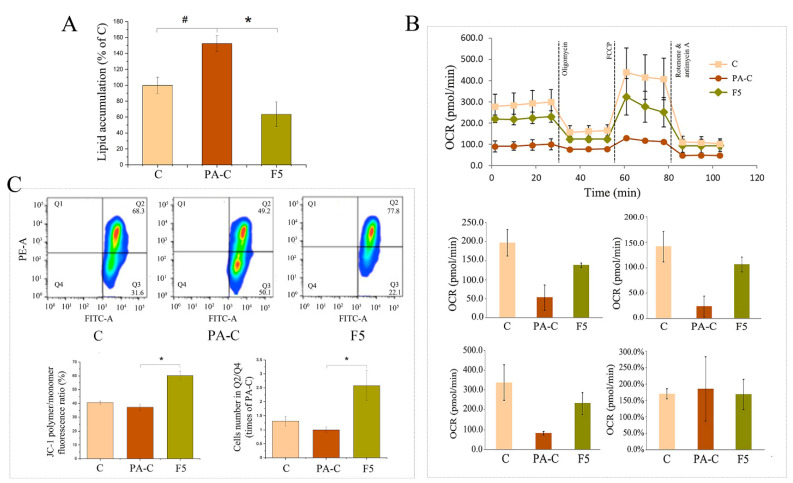
Fucoxanthin modulated lipid metabolism in HepG2 cells. (**A**) Fucoxanthin alleviated PA-induced lipid accumulation; (**B**) fucoxanthin increased mitochondrial respiration of HepG2 cells; (**C**) fucoxanthin increased mitochondrial membrane potential in HepG2 cells. Data are presented as means ± SD from three independent experiments. C: Control; PA-C: Control+ palmitate; F5: 5 μg/mL of fucoxanthin. # indicates significantly different at *p* < 0.05 compared with C. * indicates significantly different at *p* < 0.05 compared with PA-C.

Given that fucoxanthin is a principal carotenoid in NLE, pure fucoxanthin was investigated to see if it was responsible for the lipid-lowering effect of NLE in HepG2 cells. As shown in Figure 7A, fucoxanthin at a concentration of 5 µg/mL (equivalent to 100 µg/mL NLE) significantly reduced the PA-induced lipid accumulation by ~60%. Notably, the lipid content of fucoxanthin-treated cells was even lower than that of control (C group). Fucoxanthin treated cells also showed higher OCR, indicating a higher level of mitochondrial respiration (Figure 7B). Further, MMP was found to be fully reversed by fucoxanthin (Figure 7C). These data were consistent with those obtained with NLE (Figure 3, Figure 4 and Figure 5).

**Figure 8:**
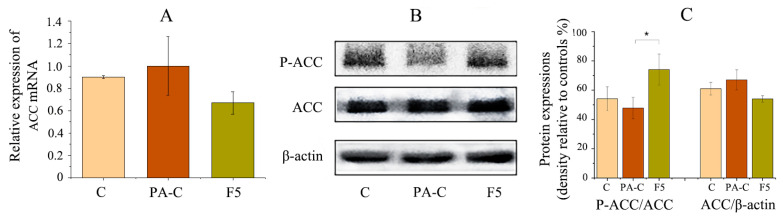
Effect of fucoxanthin on the expression of key genes and proteins associated with lipid metabolism in HepG2 cells. (**A**) Relative mRNA expression of ACC; (**B**) western blot analysis and (**C**) densitometry of immunoblots normalized to β-actin. Data are presented as means ± SD from three independent experiments. C: Control; PA-C; Control + palmitate; F5: 5 μg/mL of fucoxanthin. * indicates significantly different at *p* < 0.05 compared with PA-C.

Subsequently, expression of ACC was also investigated at mRNA and protein levels. Although no significant changes were observed for the expression of ACC at both levels (Figure 8A,B), western blotting results showed that fucoxanthin significantly upregulated the expression of p-ACC, supporting its role in attenuating the activity of fatty acid biosynthesis in HepG2 cells (Figure 8B,C). 

## 3. Discussion

The prevalence of NAFLD is increasing worldwide [13]. Accumulating evidence indicates that control of body weight, in particular, by reduction of lipid mass may help ameliorate NAFLD [14]. In the present study, NLE supplementation significantly reduced body fat and attenuated lipid accumulation in both HFD-induced C57BL/6J mice and PA-treated HepG2 cells. This was likely due to the improvement of mitochondrial lipid metabolism as evidenced by increased OCR and MMP. Suppression of fatty acid biosynthesis partly underlay the action mechanism of NLE. 

NLE is a microalgal extract containing ~5% (*w*/*w*) fucoxanthin. The lipid-lowering bioactivity of NLE is consistent with previous report that algal extract (containing 8.0 mg/g fucoxanthin) effectively reduced liver fat in NAFLD women [15]. Fucoxanthin, a dominant carotenoid in NLE, was ever reported to reduce bodyweight gain of BALB/c mice [16]. Mechanistic study in FL83B hepatocytes suggested that it could attenuate fatty acid-induced lipid accumulation through regulation of Sirt1/AMPK signaling [17]. In the current study, pure fucoxanthin at a dose equivalent to that in the NLE experiments was able to produce a similar beneficial effect against PA-induced lipid accumulation in the cells, supporting that fucoxanthin was likely the principle active component of NLE for lipid-lowering bioactivity. 

NAFLD, even for hepatic steatosis phase, is a complex metabolism disorder involving multiple mechanisms. Among all of them, mitochondrial function was one of the most vital factors for hepatic lipid metabolism. Over-accumulation of hepatic lipid and the subsequent steatohepatitis can cause proton leakage and impair mitochondrial respiratory capacity, indicating mitochondrial dysfunction [18,19]. Conversely, restoration of mitochondrial respiratory capacity may help prevent fatty acid accumulation in hepatocytes [20]. In this study, the observation that PA treatment lowered maximal respiration of the cells agrees with a report on NAFLD patients [18]. This suppression of mitochondrial respiratory capacity was dose-dependently rescued by NLE and fucoxanthin was also able to partially restore it. Moreover, both of them could reverse the attenuated basal and ATP-linked respiration caused by PA (Figure 4), indicating their capability to rescue mitochondrial function which further benefit lipid metabolism [21]. Generated by proton pumps, MMP is a widely used biomarker for functional integrity of mitochondria, especially in relation to oxidative cellular metabolism and ATP generation [22]. Lipid metabolism disorder caused by PA treatment dramatically decreased MMP of HepG2 cells, indicating damage of mitochondrial function. Notably, NLE and fucoxanthin recovered the decreased MMP caused by PA (Figure 5 and Figure 7C), indicating a restoration of mitochondrial function. These data, together with the promotion of mitochondrial respiratory capacity, reinforced the notion that maintenance of MMP could effectively induce lipid metabolism, which potentially exerts its lipid-lowering activity in hepatocytes [23]. 

ACC, facilitating the conversion of acetyl CoA to the metabolic intermediate malonyl CoA which is also an inhibitor of fatty acids oxidation, plays a central role in hepatic lipid metabolism, especially *de novo* lipogenesis that contributes to NAFLD. Besides, ACC is vital for governing the flux of carbon intermediates between carbohydrate and fatty acid metabolism [24]. Activity of ACC (usually ACC1 in human liver) could be inhibited through AMPK-mediated phosphorylation [12]. Based on this kind of mechanism, ACC began to be used as target for treatment of lipid metabolism related disease, like obesity, diabetes and NAFLD [11]. In the current study, although NLE did not temper the expression level of ACC mRNA, it significantly increased the level of phosphorylated ACC (p-ACC) relative to PA-treated cells as well as the vehicle control (Figure 6B,C), which indicates inhibition of fatty acids synthesis. This result was in line with the report that ND-654, an inhibitor of ACC, suppressed lipogenesis in animal studies and HepG2 cell lines through phosphorylation of ACC [12]. Similar results were obtained for fucoxanthin (Figure 8). In this regard, the lower lever of fatty acids accumulation by NLE/fucoxanthin in HepG2 cells could be, at least partially, mediated by inhibition of fatty acids synthesis as evidenced by down-regulation of ACC activity. Still, factors associated with lipid accumulation, like the appetite and other biochemicals involved in lipid metabolism need further investigation. The lipid-lowering bioactivity of fucoxanthin in vivo also needs to be verified in the following studies.

In summary, NLE showed lipid-lowering bioactivities in both HFD-induced steatosis mice and PA-treated HepG2 cell lines. The underlying mechanisms are likely credited to the enhancement of hepatic lipid metabolism as evidence by improved mitochondrial function and inhibition of fatty acids biosynthesis which was mediated at least in part by upregulating the phosphorylation of ACC. Fucoxanthin was likely the principal component responsible for the lipid-lowering effect of NLE. These findings suggest that NLE and other microalgal extracts rich in fucoxanthin may help prevent lipid accumulation in the liver, and thus are promising functional supplements for NAFLD patients.

## 4. Materials and Methods

### 4.1. Materials and Reagents

*Nitzschia laevis* (UTEX 2047) was cultured and harvested in our laboratory according to the method of our previous studies [7,25]. Fucoxanthin (F6932) was purchased from Sigma Chemical Co. (St. Louis, MO, USA). HepG2 cell lines were from Cell Culture Centre of Chinese Academy of Medical Sciences Peking Union Medical College (Beijing, China). DMEM medium, fetal bovine serum (FBS) and penicillin-streptomycin solution were purchased from GIBCO (Grand Island, NY, USA). Oil red, 3-(4,5-Dimethylthiazol-2-yl)-2,5-diphenyltetrazolium bromide (MTT), dimethyl sulfoxide (DMSO), paraformaldehyde and isopropanol were purchased from Sigma Chemical Co. (St. Louis, MO, USA). Sodium palmitate was purchased from Tokyo Chemical Industry (Tokyo, Japan) and fatty acid-free BSA was from Solarbio Life Sciences (Beijing, China). Mitochondrial Membrane Potential Assay Kit and RIPA Lysis Buffer were purchased from Beyotime Biotechnology (Shanghai, China). BCA Protein Assay Kit was from Bio-Rad Laboratories, Inc. (Hercules, CA, USA). Triglyceride Assay Kit was purchased from Nanjing Jiancheng Bioengineering Institute (Nanjing, China). TaKaRa MiniBEST Universal RNA Extraction Kit, PrimeScript™ RT Master Mix and TB Green™ Premix Ex Taq™ II were from Takara Bio INC. (Shiga, Japan). Antibodies against β-actin, ACC (ACC1, Available online: https://www.ncbi.nlm.nih.gov/gene/31, accessed on 5 August 2021) and phosphorylated ACC (p-ACC) were purchased from Cell Signalling Technologies (Beverly, MA, USA). The secondary antibodies (HRP-linked anti-rabbit and anti-mouse IgG) were from Abcam (Cambridge, UK). For the Seahorse XFe24 Cell Mito Stress Test, all the related reagents were obtained from Seahorse, Agilent Technologies, Inc. (Wilmington, NC, USA). All other chemicals and reagents are of analytical grade. 

### 4.2. Preparation of Nitzschia Laevis Extract and Identification of Fucoxanthin

NLE was extracted from *Nitzschia laevis* (UTEX2047) powder and its composition was measured in our previous study [7]. Fucoxanthin identification was performed using liquid chromatography-mass spectrometry (LC-MS, Waters Xevo G2QTOF, Milford, MA, USA) with an atmospheric pressure chemical ionization (APCI) and a C18 reverse phase bar (5 mm particle size, 250 mm ×4.6 mm).

### 4.3. Animals and Treatments

Five-week-old C57BL/6J mice were purchased from Vital River Laboratory Animal Technology Co. Ltd. (Beijing, China). After acclimation for one week, all mice were divided into three groups (*n* = 8): NCD (Normal chow diet, Beijing Keao Xieli Feed Co., Ltd. China), HFD (D12492, Beijing Keao Xieli Feed Co., Ltd. China) and HFD + NLE (D12492, Beijing Keao Xieli Feed Co., Ltd., Beijing, China) for 4 weeks. From the 5th week, feed mice in HFD + NLE group with 100 mg/kg/d NLE by oral gavage and the other two groups with saline solution for another 8 weeks. The mice were kept in a well-ventilated room maintained at 25 ± 2 °C with 12 h light: 12 h dark cycles. All mice were fed ad libitum. Their energy intake and bodyweight of mice were measured weekly. At the end of the experiments, all mice were sacrificed gently after blood collection by cervical dislocation. Inguinal fat and livers were isolated and weighed immediately before snap-frozen and stored at −80 °C until use. All animal related procedures were approved by the Ethics and Animal Welfare Committee of Beijing University and Technology.

### 4.4. Histological and Biochemical Analysis

All liver samples were fixed in 4% paraformaldehyde solution in PBS and embedded in paraffin. Tissue sections (5 µm) were stained with H&E and visualized under a microscope (DM2500, Leica Microsystems, Amsterdam, the Netherlands). TG content of the liver was calculated using the Triglyceride assay kit following strictly the instructions. Blood samples were immediately centrifuged at 10,000 rpm for 10 min after collection. Total cholesterol (CHO), total triacylglycerol (TG), low-density lipoprotein (LDL)-CHO, and high-density lipoprotein (HDL)-CHO and alanine aminotransferase (ALT) were measured with commercial kits (Applygen Technologies Inc., Beijing, China).

### 4.5. Cell Culture and Treatments

HepG2 cells were cultured in glucose-free DMEM supplemented with 10% FBS and 1% penicillin-streptomycin solution under 37 °C/5% CO_2_. Following serum starvation for 24 h, the cells were treated with different concentrations of NLE (10, 50, 100 µg/mL dissolved in DMSO) or fucoxanthin (5 µg/mL dissolved in DMSO) for 12 h in palmitate-supplemented medium which contained 0.5% of sodium palmitate and 1% *w*/*v* fatty acid-free BSA [26]. 

### 4.6. Cytotoxicity Assay

Cells were seeded at a density of 0.8×10^4^/well in 96-well plates. Following treatments with NLE or fucoxanthin, the medium was replaced with MTT-containing-medium (final concentration, 0.5 mg/mL) and incubated for 4 h. Sodium dodecyl sulfate (SDS) solution was added to the wells to dissolve the crystals for spectrophotometric measurement at 570 nm. Cell viability was calculated according to the absorbance of drug-treated samples relative to control. 

### 4.7. Oil Red O Staining and Quantification

Lipid content of the cells was determined by Oil red O staining [27]. Briefly, cells were washed twice with pre-chilled phosphate buffered saline (PBS) and fixed with 10% paraformaldehyde for 10 min at room temperature. The fixed cells were washed with PBS and stained with 30% Oil red O for 30 min. After staining, the cells were washed with PBS once, followed by washing with 60% isopropanol for 1 min to remove the unbound dye. Then 100% isopropanol was added, and the samples were shaken for 10 min at room temperature. Fluorescence intensity of the samples was measured at 532 nm. 

### 4.8. Mitochondrial Respiration Assay 

Consumption of exogenous and endogenous fatty acids through mitochondrial respiration was simultaneously measured using the Agilent Seahorse XF Cell Mito Stress Test (No. 103015–100), together with the XF Palmitate-BSA FAO Substrate. Briefly, HepG2 cells were seeded into the wells of an XFe24 plate, incubated for 12 h and starved for another 12 h before treatment with the drugs. OCR was evaluated in an XFe24 Extracellular Flux Analyzer (Seahorse Bioscience Inc., North Billerica, Billerica, MA, USA) with the following inhibitors: 2.5 μg/mL oligomycin, 1.6 μM FCCP and 0.5 μM antimycin A. 

### 4.9. Mitochondrial Membrane Potential Analysis

MMP was measured by using the JC-1 Mitochondrial Membrane Potential Assay Kit following the manufacturer’s guidelines. Flow cytometry analysis was conducted with a BD FACSVerse™ flow cytometer (BD Biosciences, San Jose, CA, USA).

### 4.10. mRNA Extraction and Quantitative Real-time PCR Analysis

Total RNA was extracted with the TaKaRa MiniBEST Universal RNA Extraction Kit following the manufacturer’s instructions. The cDNA was generated by using PrimeScript™ RT Master Mix. Then the genes were analysed with TB Green™ Premix Ex Taq™ II in a real-time quantitative PCR system (Bio-Rad CFX Connect, Shanghai, China). The primers used were β-actin (Forward (F): ATAGCACAGCCTGGATAGCAACGTAC; reverse (R): CACCTTCTACAATGAGCTGCGTGTG), and ACC (F: GGATGGTGTTCACTCGGTAATAGAG; R: GGGTGATATGTGCTGCGTCAT). Results were normalized to β-actin, and the relative gene expression was calculated with the 2^−ΔΔCt^ method.

### 4.11. Western Blot Analysis

Fresh cells were harvested and lysed in RIPA buffer supplemented with protease inhibitor cocktail and EDTA. Protein content was determined by a BCA protein assay kit. The lysates were resolved by sodium dodecyl sulfate polyacrylamide gel electrophoresis (SDS-PAGE) followed by transferring to polyvinylidene difluoride (PVDF) membranes. The membranes were incubated with the specified antibodies and the protein bands were visualized in an Amersham Imager 600 (GE, Boston, MA, USA) system. Quantitative analysis of the immunoblots was performed with Image J (version 1.4.3.67) software. Same procedures were applied for all proteins.

### 4.12. Statistical Analysis

All the experiments were repeated three times and the data are presented as mean ± SD. One-way analysis of variance (ANOVA) followed by Dunnett’s test was used to determine the significance of differences between groups. Statistical significance was accepted when *p* < 0.05. 

## Figures and Tables

**Table 1:** Serum parameters of mice administrated NLE.

Parameters	Units	NCD	HFD	HFD + NLE
CHO	mM	2.33 ± 0.10	4.65 ± 0.47	3.39 ± 0.13
TG	mM	0.19 ± 0.07	0.16 ± 0.05	0.13 ± 0.05
HDL-C	mM	1.33 ± 0.04	1.72 ± 0.14	1.18 ± 0.24
LDL-C	mM	0.10 ± 0.01	0.25 ± 0.03	0.2 ± 0.02
ALT	U/L	25.9 ± 3.18	127.9 ± 15.3	85.5 ± 9.32

## Data Availability

Data is contained within the article.
